# Molecular Basis of Apomixis in Plants

**DOI:** 10.3390/genes12040576

**Published:** 2021-04-16

**Authors:** Diego Hojsgaard

**Affiliations:** Department of Systematics, Biodiversity and Evolution of Plants, Albrecht-von-Haller Institute for Plant Sciences, University of Göttingen, Untere Karspüle 2, 37073 Göttingen, Germany; diego.hojsgaard@biologie.uni-goettingen.de

Sexual reproduction in plants is a complex, stringently regulated process that leads to the creation of diaspores for a new generation: sexual seeds. Traditionally, sexuality is exploited to segregate or selectively assemble desired genes and traits during the creation of new crop varieties. However, the exploitation of sexuality also imposes constraints on plant breeding, which include high seed costs and time-consuming methods. Most of these limitations can be mitigated by sequentially exploiting sexuality and apomixis during plant breeding.

Apomixis is the consequence of a concerted mechanism that harnesses the sexual machinery and acts in a way that coordinates developmental steps in the ovule to produce an asexual (clonal) seed. Altered sexual developments involve widely characterized functional and anatomical changes in the meiosis, gametogenesis and embryo and endosperm formation. The ovules of apomictic plants skip meiosis and form unreduced female gametophytes whose egg cells develop into a parthenogenetic embryo, and the central cells may or may not fuse to a sperm to develop the seed endosperm. Thus, functional apomixis involves at least three components, apomeiosis + parthenogenesis + endosperm development, modified from sexual reproduction that have to be coordinated at the molecular level to progress through the developmental steps and form a clonal seed. Despite recent progress uncovering specific genes related to apomixis-like phenotypes and clonal seed formation, the molecular basis and regulatory network of apomixis is still unknown. This is a central problem underlying the current limitations of apomixis breeding.

This Special Issue collects twelve publications addressing different topics around the molecular basis of apomixis, illustrating recent discoveries and advances towards understanding the genetic regulation of the trait and discussing the possible origin of apomixis and the remaining challenges for its commercial deployment in plants.

Since theories that apomixis is a phenomenon based on gain- or loss-of-function mutations over sexuality remain open, Barcaccia et al. [1] reassess the evolutionary origin of apomixis in angiosperms and their alternative developmental pathways and present phylogenetic and genetic evidence supporting that apomixis evolved from sex and is a consequence of the molecular disruption of key players in sexual development. Furthermore, Schmidt [2] gives an overview of the molecular aspects of apomixis in higher plants and provides a clear interpretation of the regulatory complexity involved in apomictic development, emphasizing the active role of DNA- and RNA-binding proteins, as well as non-coding RNAs, in the activation and repression of developmental programs through epigenetic regulatory mechanisms. Similarly, Ortiz et al. [3] summarize the vast information on apomixis in a case study on *Paspalum* spp. and provide details on the key aspects of apomictic development in the genus and the various genetic analyses used, including the molecular characterization of genomic loci, functional characterization of three reproductive candidate genes (*ORC3*, *QGJ* and *TGS1*) and a roadmap for further genome-based studies.

Further molecular details on apomixis have been derived from different plant species. Mateo de Arias et al. [4] use genetic and cytoembryological analyses combined with stress treatments in five species to present substantial evidence in support of a polyphenic condition for meiosis and apomeiosis determined by metabolic states. The study lays the groundwork for a new avenue of research and future studies focused on integrating metabolism into the current understanding of apomixis and on unraveling the role that metabolic homeostasis plays in reproductive switches. Likewise, Selva et al. [5] use gene expression and cytoembryological analyses combined with water stress experiments to pinpoint genes that modulate the expression of sexuality in the apomictic grass species *Eragrostis curvula*, and to identify genes with more specific roles in the stress response and control of the reproductive mode. Pellino et al. [6] take advantage of gene expression analyses on microdissected ovules to disclose genes that are differentially expressed between sexual and apomictic individuals of the *Ranunculus auricomus* complex, owing to effects of transgressive gene expression, parent of origin and ploidy. Three genes probably involved in the reproductive steps are highlighted. In *Poa pratensis*, Marconi et al. [7] use detailed genetic and in situ hybridization analyses, as well as protein prediction and molecular modelling tools, to characterize a collection of apomixis-related alleles identified in relation to *APOSTART*, the putative gene with an active role in the development of apomixis in this grass species.

Parthenogenesis is a central component of apomixis and is often associated with the development of the endosperm, the least studied feature in apomictic plants. van Dijk et al. [8] collect cytoembryology, flow cytometry and molecular marker data from experimental crosses in *Taraxacum officinale* and advance the genetic characterization of autonomous endosperm formation in the Asteraceae by providing evidence supporting a three-locus model for apomixis, with parthenogenesis and diplospory controlled by independent dominant loci and autonomous endosperm development under complex regulatory control. Focusing on parthenogenesis, Zhang et al. [9] take a further step toward inducing apomixis in sexual plants. These colleagues transferred the *PsASGR-BABYBOOM*-like gene (*PsASGR-BBML*) that confers parthenogenesis in the apomict grass *Pennisetum squamulatum* (and other monocot species) into tobacco plants and confirmed, for the first time, that the *PsASGR-BBML* gene regulated by egg cell-specific promoters enables parthenogenesis in a dicotyledonous species. Another substantial advance is derived from Henderson et al. [10], who introduced a *Cas9* construct in an apomictic *Hieracium* species via *Agrobacterium*-mediated leaf disk transformation and evaluated the efficiency of CRISPR/Cas9 editing to target the endogenous *PHYTOENE DESATURASE* (*PDS*) gene. The study—featured in this collection—demonstrates, for the first time, that gene editing tools can be effectively used in apomixis research and inaugurates a new stage for the identification of apomixis genes.

Finally, an opinion piece summarizes the relevant molecular data and the three currently accepted but divergent hypotheses explaining the nature of apomixis to point to a probable single molecular event with a multigenic effect on reproductive development implicated in the origin of apomixis, remarking on the need to find a unifying molecular model to fully exploit apomixis technology [11]. In addition, Scheben and Hojsgaard [12] discuss alternative methods from the gene-editing toolbox and their feasible use to induce apomixis in sexual plants depending on the type of molecular model of the genetic control of apomixis that is chosen, and stress the importance of understanding the molecular basis of apomixis and its natural variation in apomictic plants for trait breeding and optimization in sexual ones.

Each of the plant species used as model systems in apomixis research has its own characteristics and challenges. The present collection covers plant species that exhibit diverse changes in the sexual machinery and a range of methodologies, from embryology and genomics to genetic modification and bioinformatics methods, that exemplify such particularities and expose the efforts and ingenuity that researchers devote to uncovering the molecular details of apomixis in plants. Each of the contributions, many of which point to new research directions, paves the way toward a better understanding of apomixis and guides the advancement of this scientific topic by bringing us closer to resolving the molecular basis of apomixis and its biotechnological application.
